# Spices Volatilomic Fingerprinting—A Comprehensive Approach to Explore Its Authentication and Bioactive Properties

**DOI:** 10.3390/molecules27196403

**Published:** 2022-09-28

**Authors:** Sergio Izcara, Rosa Perestrelo, Sonia Morante-Zarcero, Isabel Sierra, José S. Câmara

**Affiliations:** 1 Departamento de Tecnología Química y Ambiental, Escuela Superior de Ciencias Experimentales y Tecnología, Universidad Rey Juan Carlos, C/Tulipán s/n, 28933 Móstoles, Madrid, Spain; 2 CQM—Centro de Química da Madeira, Universidade da Madeira, Campus da Penteada, 9020-105 Funchal, Portugal; 3 Departamento de Química, Faculdade de Ciências Exatas e Engenharia, Universidade da Madeira, Campus da Penteada, 9020-105 Funchal, Portugal

**Keywords:** authenticity, bioactive properties, chemometrics, fingerprint, HS-SPME/GC-MS, spices, volatile organic metabolites

## Abstract

Volatile organic metabolites (VOMs) present in different spices can provide distinct analytical biosignatures related to organoleptic properties and health benefits. This study aimed to establish the volatilomic fingerprint of six of the most consumed spices all over the world (saffron (*Crocus sativus* L.), cinnamon (*Cinnamomum verum*), cumin (*Cuminum cyminum* L.), black pepper, (*Piper nigrum* L.), sweet paprika (*Capsicum annuum* L.), and curry (a mix of different herbs and spices)). Based on headspace solid phase microextraction (HS-SPME) followed by gas chromatography-mass spectrometry (GC-MS) analysis, this is a powerful strategy to explore and establish the spice’s volatile pattern and unravel the potential health benefits related to the most important VOMs identified in each spice. This comprehensive knowledge will help in the definition of their authenticity, while simultaneously protecting against potential frauds and adulterations. A total of 162 VOMs were identified. Semi-quantitative assessments revealed that terpenoids and sesquiterpenoids amounted to the major volatile class in the investigated spices, except for cinnamon, where carbonyl compounds are the major group. Most of the studied spices comprised key characteristics of aroma and health bioactive compounds, e.g., dihydrojuneol in saffron, cinnamaldehyde in cinnamon, cuminaldehyde in cumin and curry, and caryophyllene in black pepper. The principal component analysis (PCA) and partial least-squares discriminant analysis (PLS-DA) successfully discriminated the investigated spices, being α-cubebene, 3-methyl butanal, β-patchoulene and β-selinene, the most important VOMs (highest VIP’s) that contributed to its discrimination. Moreover, some VOMs have a high influence on the spice’s bioactive potential, helping to prevent certain diseases including cancer, inflammatory-related diseases, diabetes, and cardiovascular diseases.

## 1. Introduction

Since antiquity, spices have been used worldwide for a variety of purposes. From natural flavoring and preservative agents in beverages, pharmaceuticals, and foods, to ingredients in cosmetics and perfumes, to their use as preventive agents with potential benefits in human health [1,2,3].

Its composition includes the presence of natural phytochemicals such as polyphenols, volatile organic metabolites (VOMs), vitamins and sulfur-containing compounds, among others. These natural phytochemicals, together with the complementary actions they can exert on human health and their bioactive potential (antioxidant, antimicrobial, antiviral, anti-inflammatory, antidiabetic, anti-obesity, anti-pyretic, antihypertensive, and antidepressant effects, cytochrome and other enzyme inducers, reducing induction and advancement of cancer cell development, and cardio and neuroprotective effects), helps to explain the effects of spices and other rich foods in bioactive constituents on the human metabolism [4,5]. For example, capsaicin has been shown to reduce reactive oxygen species (ROS) and thereby inflammation. Oleoresin from rosemary exerts potent antioxidant properties, retards the development of an off-flavor, and inhibits oxidative rancidity in some products [1,2,3,4,5]. In addition, some constituents of spices, herbs, and plant-origin foods, including lycopene, curcumin, carvone, and limonene, are associated with a reduced risk of cancer [3,4,5]. This outstanding potential has boosted the increasing interest among the scientific community in spices and other plant-based foods with a high content of bioactive compounds. Moreover, the increasing demand for flavors from natural sources gives spices enormous potential in this context.

Saffron (*Crocus sativus* L.), cinnamon (*Cinnamomum verum*), cumin (*Cuminum cyminum* L.), black pepper (*Piper nigrum* L.), sweet paprika (*Capsicum annuum* L.), and curry belong to the group of spices most consumed all over the world. Saffron, the dried red stigma of flowers of *Crocus sativus* L., is mainly produced by Iran, followed by India, Afghanistan, Morocco, and Euro-Mediterranean countries including Greece, Spain, and Italy [6]. It is a highly valued spice not only for its unique aroma, taste, and color, but also because the harvesting and separation of the stigmas are done manually, being tedious and labor-intensive work. Although mainly valued as an additive for its property of flavoring, coloring, and tasting food, saffron has been used traditionally in alternative systems of medicine for various diseases due to its pharmacological properties, such as antioxidant, antidepressant and anticarcinogenic [6]. It is even able to act as an anti-inflammatory and antiviral agent in the prevention of severe symptoms of COVID-19 [7]. Crocin, picrocrocin and safranal are the main secondary metabolites of saffron, which are responsible for the color, bitter taste, and odor, respectively [8].

Belonging to the Lauraceae family, *Cinnamomum verum* (cinnamon) is a widely used spice due mainly to its medicinal and culinary applications. It is native to Sri Lanka and southern India and has traditionally been used to treat several diseases and ailments. Cinnamaldehyde is the characteristic compound of cinnamon and is primarily responsible for rendering taste, odor and flavor to foodstuffs [9]. However, compounds such as eugenol, caryophyllene, cinnamyl acetate and cinnamic acid are also found in high amounts, being responsible for the numerous pharmacological activities of this spice, including antioxidant, antimicrobial, anti-inflammatory, anticancer, antidiabetic, wound healing, and anti-HIV, among others [4].

Cumin (*Cuminum cyminum* L.) belongs to the Apiaceae family and is a thin and aromatic annual plant, which is indigenous to southwest Asia and the eastern Mediterranean region (Egypt), with India as the main producer. It is a very versatile spice with numerous attributes. It is commonly used in food flavoring and perfumery, but also traditionally in the treatment of dyspepsia, diarrhea, abdominal colic and jaundice. This is due to its excellent pharmacological attributes, including anti-platelet aggregation, hypoglycemic, antioxidant, antidiabetic, anti-inflammatory, and anticancer effects [10]. Cuminaldehyde is the main bioactive compound of cumin [11], although it also contains other VOMs such as cymene, cuminic alcohol (cuminol), γ-terpinene, safranal, limonene, eugenol, β-myrcene, α-phellandrene, β-phellandrene, α- and β-pinene [12], which are probably responsible for the numerous pharmacological activities that cumin possesses.

*Piper nigrum* L. (black pepper) belongs to the Piperaceae family and is one of the oldest and most extensively used spices, largely cultivated in the southwestern part of India and other tropical regions. Beyond its culinary uses, black pepper has been used for treating asthma, respiratory tract infections, and rheumatoid arthritis [9]. In addition, some studies have reported its use in gastrointestinal diseases [13] and as an anticancer agent [14]. The most prominent active chemical constituents of black pepper include piperidine, piperine, limonene, α-pinene, β-pinene and camphene [15].

Sweet paprika (*Capsicum annuum* L.) is also one of the most widely consumed spices worldwide. It consists of the dried and ground ripe fruits of *C. annuum,* a non-pungent analogue of capsaicin belonging to the family of capsinoids. It constitutes a natural source of different polyphenols, ascorbic acid, and carotenoids, which play a key role in preventing certain diseases including cardiovascular diseases and some kinds of cancer [16,17]. In addition, it possesses other bioactive compounds (capsaicin, curcumin, tocopherol, lutein, carotene, capsanthin, and quercetin) with potential antioxidant and anti-inflammatory effects [13,16,17].

Curry is a mixture of different herbs and spices, including chili, basil, fennel, celery, saffron, cinnamon, cardamom, dried onion, coriander, cloves, cumin, turmeric, fenugreek, ginger, mustard, nutmeg, pepper, cayenne or tamarind. Currently, curry is used in cuisines all over the world, with its origins being in the Asian continent. Its composition can vary, which is why curry can contain several bioactive compounds. However, turmeric is usually found in large proportions in the composition of curry, where curcumin is the main bioactive compound. In addition to conferring the typical yellow color, curcumin is responsible for the various pharmacological effects that help to fight against some cardiovascular diseases and cancer [13,15].

The extremely high market prices of most spices (for instance, saffron can reach 50 M €/Kg), increases the adulteration susceptibility of the original product. In addition, due to its nutritional value, high demand, and associated potential health benefits, a comprehensive insight into the characteristic secondary metabolites responsible for its authenticity and quality is of paramount importance as a powerful strategy for the prevention and detection of potentially fraudulent activities. In this context, gas chromatography-mass spectrometry (GC-MS) is considered the gold standard instrumental technique for VOMs analysis in a wide range of samples [18]. However, the previous sample preparation is fundamental in concentrating the VOMs and removing interferences, particularly from complex samples, as food matrices [19]. Several conventional analytical extraction techniques, such as solid-liquid extraction (hydro-distillation), supercritical fluid extraction (SFE), Soxhlet and others, can be employed for the extraction and pre-concentration of the VOMs from food matrices. However, these techniques present some drawbacks since they require significant amounts of organic solvents and samples, they are time-consuming and require exhaustive concentration steps, which can generate artefacts leading to an inaccurate elucidation of the volatiles [20]. In this context, headspace solid-phase microextraction (HS-SPME) is a well-established technique that has been successfully applied in the characterization of the volatile profile of new fruits and vegetables [21,22]. This technique requires relatively short extraction times and minimal sample handling, combining extraction and concentration in a single step. In addition, it does not require the use of potentially harmful organic solvents, making it an environmentally and analyst-friendly extraction technique. Moreover, its combination with GC coupled to a mass spectrometer with a quadrupole inert mass selective detector (qMS) retrieves high sensitivity, reproducibility, and robustness. In addition, a few studies applied multivariate statistical data analysis to chromatographic data sets as a powerful and fast strategy to obtain volatile fingerprints and discriminate between the different food matrices [21,22,23].

Therefore, the main purpose of this work was to establish the comprehensive volatilomic profile of five of the most consumed spices worldwide, namely saffron, cinnamon, cumin, black pepper and sweet paprika, in addition to curry, using HS-SPME/GC-qMS to examine the dominant VOMs and evaluate the diversity and similarity in the VOMs patterns. The definition of the volatilome of each spice will allow for a better understanding of its ability in authenticity determination and quality control, and therefore the detection of potential adulterations which affect the genuine volatile profile. The combination of the chromatographic data set with multivariate statistical data analysis (MSDA) was also used to extract useful information concerning the spice’s quality and authenticity. This approach provides insights into understanding the chemistry behind the bioactive and flavor properties of these spices, consequently providing consumers with quality and safety guarantees.

## 2. Results and Discussion

### 2.1. Volatomic Fingerprinting from Spices

Due to high demand, nutritional value, and the *sui generis* typical aroma highly appreciated by consumers, a volatilomic-based analytical approach was used to establish the volatilomic fingerprint of each spice as a useful strategy to assess and define its quality and authenticity. Figure S1 (Supplementary Material) shows the typical chromatograms of the investigated spices, obtained by HS-SPME_DVB/CAR/PDMS_/GC-qMS. Their chromatographic profiles show that the concentration range of the volatile compounds differs enormously for the various spices. For instance, black pepper presents a very complex chromatogram with most volatiles at high concentrations (based on peak area intensity), whereas the volatile compounds identified in sweet paprika shows relatively low concentrations (Figure S1).

**Figure 1:**
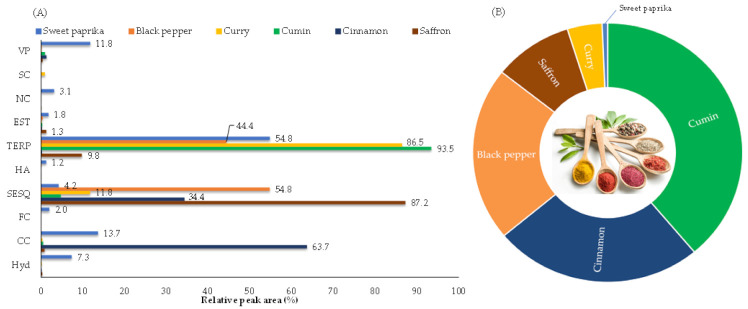
(**A**): Distribution of the identified chemical classes according to spice; and (**B**): Distribution of the total volatile fraction for each investigated spice. VP: Volatile phenols; SC: Sulfur compounds; NC: Nitrogen compounds; EST: Esters; TERP: Terpenoids; A: Alcohols; SESQ: Sesquiterpenoids; FC: Furanic compounds; CC: Carbonyl compounds; Hyd: Hydrocarbons.

In total, 162 VOMs were identified, most of them in black pepper (**69**), while in other spices the number of identified VOMs ranged from 49 (cumin) to 59 (cinnamon). The identified VOMs belong to several chemical groups including sesquiterpenoids (**66**), terpenoids (**48**), carbonyl compounds (**14**), and hydrocarbons (**12**). At a lower extent were identified eight esters, six volatile phenols, three furanic compounds, two higher alcohols, two nitrogen compounds and one sulfur compound (Figure 1A). Figure 1B shows the distribution of the total volatile fraction for each investigated spice.

The sui generis character of spices and other foods is influenced by the composition, the presence of key aroma compounds, and concentrations which can determine the kind of aroma perceived [24]. The major VOMs chemical class in the current study (Figure 1A) is the class of terpenoids, which are widely produced in the plant kingdom [25], followed by sesquiterpenoids, and to a lower extent by carbonyl compounds.

The predominant VOMs present in the top 10 investigated spices include α-curcumene and dihydrojuneol in saffron; cinnamaldehyde and copaene in cinnamon; cuminaldehyde and γ-terpinene in cumin; caryophyllene and 3-carene in black pepper; *p*-cymene and γ-terpinene in sweet paprika; and cuminaldehyde and linalool in curry.

The detailed list of all VOMs identified in the analyzed spices and their respective obtained experimental data, including retention times, KIs, molecular formula (MF), chemical families, and relative peak area, is shown in Table 1.

Of the 162 VOMs identified, only eight are common to all the spices analyzed—α- and β-pinene, limonene, *p*-cymene, copaene, caryophyllene, β-bisabolene, and cuminaldehyde. On the other hand, some VOMs, described in Table S1, were identified only in a certain type of spice (Table S1).

### 2.2. Saffron

A total of 56 VOMs were identified in saffron (*C. sativus* L.), being mainly characterized by sesquiterpenoids (87.2%), followed by terpenoids (9.8%) (Figure 1A; Table 1). The most dominant VOMs identified in saffron include dihydrojuneol (#161), α-curcumene (#129), β-sesquiphellandrene (#128), (-)-δ-cadinene (#115) and β-bisabolene (#117), representing about 78.8% of its total volatile profile. VOMs such as caryophyllene isomer (#87), α-phellandrene (#28), *p*-cymene (#42) and epizonarene (#104), were also identified, although in lower amounts (relative peak areas of 2.89%, 2.85%, 1.83% and 1.75%, respectively) (Table 1). The VOMs identified only in saffron are listed in Table S1. Cozzolino et al. [27] conducted a comprehensive evaluation of the volatomic fingerprint of saffron with the aim of testing its authenticity and quality. Many of the VOMs identified in our samples agrees with the data provided in that work.

### 2.3. Cinnamon

Fifty-seven VOMs were identified in cinnamon (*C. verum*). Carbonyl compounds (63.7%) and sesquiterpenoids (34.4%) are the major chemical groups determined in the cinnamon samples analyzed, representing 98.1% of its total volatile fraction (Figure 1A; Table 1). Such abundance is mainly provided by the flavonoid cinnamaldehyde (#150), the key aroma compound and the most dominant in cinnamon, followed by copaene (#62), δ-cadinene (#126), α-muurolene (#118) and calamenene (#138). These are the most abundant VOMs, in terms of the relative peak area, identified in the cinnamon samples, representing 86.1% of its total volatile profile. Other VOMs were also identified, although with a lower contribution, such as γ-muurolene (#108), methyl eugenol (#148), α-calacorene (#142), α-curcumene (#129) and bicyclogermacrene (#114) (relative peak areas of 2.02%, 1.31%, 0.84%, 0.72% and 0.72%, respectively) (Table 1). The VOMs identified only in cinnamon are listed in Table S1. Some previous studies carried out on cinnamon [4,28] agree with the data obtained in this work.

### 2.4. Cumin

A total of 49 VOMs were identified in cumin (*C. cyminum* L.), where the chemical groups of terpenoids (93.5%) and sesquiterpenoids (4.7%) contributed mainly to the total volatile profile of these spices, representing over 98.1% of its total volatile fraction (Figure 1A; Table 1). Thus, the most abundant VOMs in the analyzed cumin samples are cuminaldehyde (#130), γ-terpinene (#36), β-pinene (#22), *p*-cymene (#42) and pinocamphone (#78), with relative peak areas of 55.5%, 16.2%, 8.2%, 6.5% and 3.0%, respectively (representing over 89.3% of the total volatile profile). In smaller quantities, γ-eudesmol (#153), methyl eugenol (#148), and β-myrcene (#27) were also identified, with relative peak areas of 2.23%, 0.69%, and 0.65%, respectively (Table 1). The VOMs identified only in cumin are listed in Table S1. Previous studies carried out on cumin [10,12,28] confirm the data obtained in this work.

### 2.5. Curry

Curry is a mixture of different spices, including chili, basil, fennel, celery, saffron, cinnamon, cardamom, dried onion, coriander, cloves, cumin, turmeric, fenugreek, ginger, mustard, nutmeg, pepper, cayenne, or tamarind. A total of 53 VOMs were identified in curry, where the chemical groups of terpenoids (86.5%) and sesquiterpenoids (11.8%) contributed mainly to the total volatile profile of curry, representing over 98.2% of its total volatile fraction (Figure 1A; Table 1). In this sense, the major VOMs identified in the curry sample include cuminaldehyde (#130), linalool (#68), γ-terpinene (#39), *p*-cymene (#42) and dihydrojuneol (#161), with relative peak areas of 41.5%, 23.8%, 6.1%, 5.9% and 4.9%, respectively (representing over 82.2% of its total volatile profile). In smaller quantities, the VOMs like β-pinene (#22), α-curcumene (#129), citronellol (#125), γ-eudesmol (#153) and camphor (#65) were also identified, with relative peak areas of 2.14%, 1.45%, 1.44%, 1.42% and 1.26, respectively (Table 1). The VOMs identified only in curry are listed in Table S1. Similar results were obtained in previous studies [10,13].

### 2.6. Black Pepper

Black pepper (*P. nigrum* L.) is slightly more aromatic than the other spices investigated, agreeing with its volatile profile containing sixty-nine VOMs. Sesquiterpenoids (54.8%) and terpenoids (44.4%) are the more abundant chemical groups found in the black pepper samples analyzed, representing 99.2% of their total volatile fraction (Figure 1A; Table 1). Such abundance is mainly provided by caryophyllene isomer (#87), followed by (+)-3-carene (#25), limonene (#31), δ-elemene (#57), β-pinene (#22) and copaene (#62). These are the most abundant VOMs, in terms of the relative peak area, identified in the black pepper samples, representing over 73.1% of its total volatile profile. Other VOMs were also identified, although with a lower contribution, such as α-phellandrene (#28), epizonarene (#104), *p*-cymene (#42), δ-cadinene (#126) and β-myrcene (#27) (relative peak areas of 2.77%, 2.71%, 2.40%, 2.02% and 1.99%, respectively) (Table 1). The VOMs identified only in black pepper are listed in Table S1. Some published studies show the agreement of the identified VOMs in the black pepper samples analyzed [13,29,30].

### 2.7. Sweet Paprika

**Figure 2:**
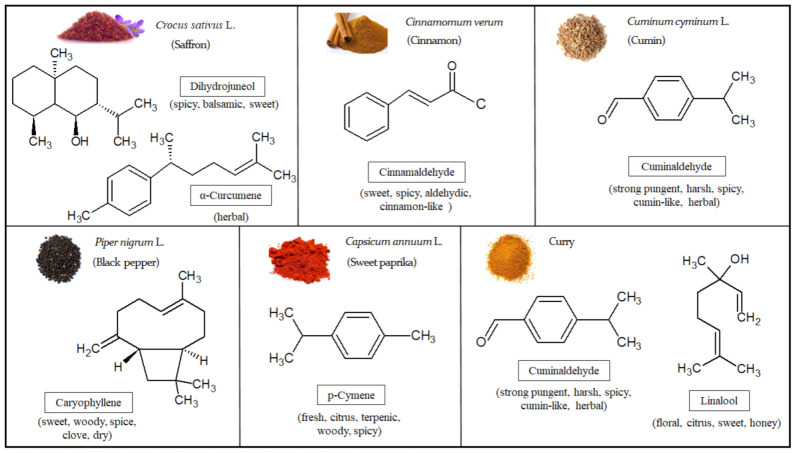
Chemical structures and characteristic odor associated with the major volatile organic metabolites (VOMs) identified in the investigated spices.

A total of 50 VOMs were identified in sweet paprika (*C. annuum* L.), being mainly characterized by terpenoids (54.8%), followed by carbonyl compounds (13.7%) and volatile phenols (11.8%) (Figure 1A; Table 1). The principal VOMs identified in sweet paprika include *p*-cymene (#42), γ-terpinene (#36), eugenol (#155), limonene (#31), thymol (#156), cuminaldehyde (#130), cinnamaldehyde (#150) and geraniol (#135), representing over 41.7% of its total volatile profile. In this sense, the sweet paprika sample is richer than the other spices, since many VOMs contribute to its volatile composition with very similar relative peak areas. On the other hand, VOMs such as benzaldehyde (#67), β-pinene (#22), hexanal (#20), 1-methyl pyrrole (#26) and caryophyllene isomer (#87) were also identified, although in lower amounts (relative peak areas of 2.92%, 2.64%, 2.59%, 2.52% and 2.13%, respectively) (Table 1). The VOMs identified only in sweet paprika are listed in Table S1. Kevrešan et al. [31] confirm the presence of some VOMs identified in the sweet paprika samples analyzed in this investigation. The chemical structures of the most representative VOMs identified in each investigated spice and the corresponding odor descriptors are presented in Figure 2.

### 2.8. Bioactive Potential of VOMs Identified in the Investigated Spices

It is well known that the spices investigated in this work have long been used to add flavor to the foodstuffs and to enhance the quality of the food. In addition, many of them also act as excellent preservatives which increase the shelf life of foods by delaying the spoilage process [26]. Such spices are also a rich source of biologically active compounds, which have antioxidant, antimicrobial, anti-inflammatory, antidiabetic, cytotoxic, antiproliferative, anticancer and even anti-HIV properties, among others [4,13,32]. These properties are beneficial to human health and help to fight several ailments of the human body.

The VOMs are part of such biologically active compounds and have a high influence on the spice’s bioactive potential. That is why many of the VOMs identified in the spices analyzed have been previously reported in other food samples, with a significant number of biological activities such as those mentioned above [33,34]. Therefore, these VOMs could help prevent and treat diseases like cancer [35], inflammatory diseases [36], diabetes [37], and cardiovascular diseases [38], among others. Table 2 shows the potential bioactive effects of some VOMs identified in the spice samples under investigation.

Among these VOMs, terpenoids are one of the most dominant chemical families found in fruits and vegetables [39], which agrees with the results obtained in this work. Terpenoids, biosynthesized through isopentyl diphosphate, and the methylerythritol phosphate and mevalonate pathways are the major contributors to the total volatile composition of cumin, curry and sweet paprika (Figure 1A). In turn, sesquiterpenoids are the major contributors to the total volatile composition of saffron and black pepper and are abundantly present in the total volatile fraction of cinnamon, where carbonyl compounds are more abundant (Figure 1A).

Therefore, terpenoids and sesquiterpenoids are the ones that contributed most to the characterization of the total volatile profile of the spices investigated. Monoterpenes have several beneficial biological effects reported in several works [36,41]. Terpenoids, such as α-pinene (#15), sabinene (#23), (+)-3-carene (#25), β-myrcene (#27), α-phellandrene (#28), α-terpinene (#30), limonene (#31), β-phellandrene (#32), *p*-cymene (#42), linalool (#68), α-terpineol (#107), cuminaldehyde (#130) and thymol (#156), possess high potential antioxidant, antimicrobial, antibacterial, anti-inflammatory, antidiabetic, neuroprotective and immunostimulant properties [10,36,38,41,42]. However, the most remarkable bioactivity of terpenoids is related to their anticancer potential, acting at different stages of tumor development and in different mechanisms of action (inhibition, regulation of intracellular signaling pathways) [42,43]. Limonene was the third and fourth major VOM, in terms of relative peak area, found in the black pepper and sweet paprika samples, respectively. The chemopreventive and chemotherapeutic properties of limonene against human cancers were widely demonstrated by Paduch et al. [44] and Kris-Etherton et al. [45]. Cuminaldehyde is an oxidized aldehyde monoterpene found as a major VOM in the cumin and curry spices analyzed. The relative peak area obtained for this compound in the cumin sample was very remarkable (9694.8), being more than twelve times greater than that determined in the curry sample (Table 1). Cuminaldehyde was also identified in the sweet paprika sample, although in lower amounts. Therefore, the results obtained about cuminaldehyde agree with those reported by Ebada [11], with cuminaldehyde being the main bioactive compound of cumin and presenting many of the bioactive properties cited previously [10]. On the other hand, several sesquiterpenoids also stood out in the volatile composition of some of the spices investigated. For example, dihydrojuneol (#161) and α-curcumene (#129) were the most abundant VOMs, in terms of relative peak areas, in the saffron sample, and caryophyllene isomer (#87), in black pepper (Table 1). Even though in smaller relative peak areas, dihydrojuneol was also found in curry, α-curcumene was identified in the cinnamon and curry samples, and caryophyllene isomer was also present in the volatile profile of saffron and sweet paprika (Table 1). Even though they were not the most abundant VOMs in the cinnamon sample, the sesquiterpenoids copaene (#62), δ-cadinene (#126), and α-muurolene (#118) were identified in notable quantities, representing over 20% of the total volatile profile of this spice (Table 1).

Allyl isothiocyanate (#49), belonging to the chemical family of organosulfur compounds, was identified in the analyzed curry sample. These compounds are highly reactive phytochemical metabolites and are usually present in the composition of cruciferous vegetables [46]. In addition, they also have interesting properties and bioactivities, mainly antibacterial, antiproliferative, cytotoxic and anticancer effects.

On the other hand, carbonyl compounds are present in all the analyzed spices, in greater or lesser amounts, but standing out above all in the cinnamon sample, where the contribution of carbonyl compounds to the total volatile profile was higher than 63%. Cinnamaldehyde (#150) was the most prominent carbonyl compound identified in cinnamon and, although in smaller amounts, was also identified in cumin. In the case of the cinnamon sample, the amount of cinnamaldehyde was very remarkable (6996.9 relative peak area), being 83 times the amount of cinnamaldehyde present in the cumin sample (Table 1). This result obtained in our work agrees with that stated by Singh et al. [4] in their research, where an extensive review of the phytochemical and pharmacological properties (antimicrobial, antioxidant, anti-inflammatory, anticancer, antidiabetic, anti-HIV, among others) of cinnamon is carried out, concluding that cinnamaldehyde is a main and characteristic VOM of cinnamon, and is also responsible for its typical aroma.

### 2.9. Multivariate Statistical Data Analysis. Characterization of Spices

**Figure 3:**
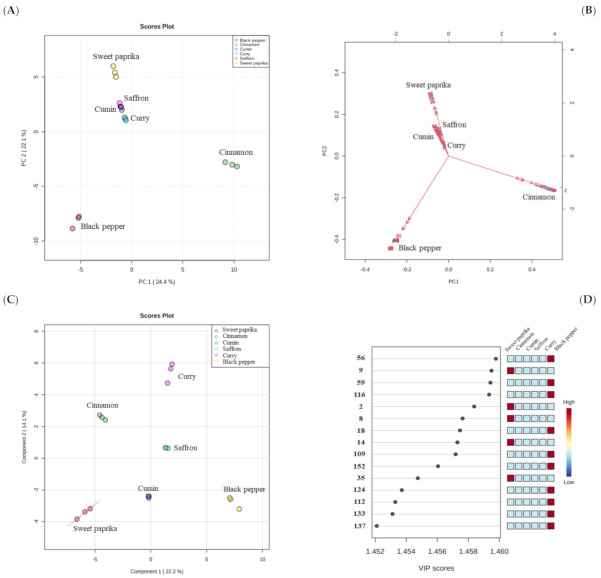
Multivariate statistical analysis (MVSA) using principal component analysis (PCA) and partial least square-discrimination analysis (PLS-DA) of the volatile signature of spices. (**A**) PCA score plot and (**B**) biplot. (**C**) PLS-DA score plot and (**D**) selected volatile organic metabolites (VOMs) based on variable importance in the projection (VIP) score contributing to the variance observed in the PLS-DA model. The numbers in the VIP graph are described in Table 1.

To evaluate the ability of HS-SPME/GC–qMS data to characterize spices in terms of their volatilomic fingerprint, a statistical analysis of the chromatographic data matrix was performed using the MetaboAnalyst 5.0 web-based tool [30]. The PCA and PLS-DA were applied as multivariate analyses (Section 2.5). The PCA is an unsupervised method that was performed to visualize the difference/similarity among sample profiles and detect significant variables (i.e., VOMs) that contribute to these discrepancies. Figure 3A,B shows the PCA score plot and biplot from the analyzed spices. The variances of the first and second principal components (PC1 and PC2) were 24.4 and 22.1%, respectively, representing 46.5% of the total VOMs variability of data, allowing a good differentiation of the spices, except for saffron and cumin.

Black pepper, projected in PC1 and PC2 negative, was chiefly characterized by β-pinene (#22), (+)-3-carene (#25), α-phellandrene (#28) and copaene (#62), whereas cinnamon samples were placed in the PC1 positive quadrant and PC2 negative quadrant and were characterized by α-muurolene (#118), calamenene (#138), α-calacorene (#142) and cinnamaldehyde (#150). The rest of the spices involved in the study (curry, sweet paprika, cumin and saffron) were projected in PC1 negative quadrant and PC2 positive quadrant. Curry was characterized by *p*-cymene (#42), citronellol (#125), cuminaldehyde (#130) and dihydrojuneol (#161). On the other hand, the samples of sweet paprika were characterized by γ-terpinene (#36) and eugenol (#155). The VOMs responsible for the characterization of the cumin samples were *p*-cymene (#42), pinocamphone (#78) and cuminaldehyde (#130). Finally, saffron was characterized by (-)-δ-cadinene (#115), β-sesquiphellandrene (#128) and dihydrojuneol (#161). PLS-DA was used as a supervised clustering method and revealed differentiation among spices (Figure 3C). A total variance of 36.3% was obtained by the first two principal components obtained from PLS-DA. Furthermore, 15 differently expressed VOMs were found with a presented VIP score ≥ 1.45, being the most relevant compounds and having the greatest discriminatory power to characterize the six spices studied (Figure 3D). Among these 15 significant VOMs, six sesquiterpenoids (α-cubebene (#56), β-patchoulene (#59), β-selinene (#116), β-cadinene (#109), germacrene B (#124) and valencene (#112)), two carbonyl compounds (3-methyl-butanal (#9) and 2-methyl-butanal (#8)), two hydrocarbons (2,4-dimethylheptane (#2) and 4-methyl decane (#14)), two esters (methyl salicylate (133) and thymol acetate (137)), one terpenoid (α-fenchene (#18)), one volatile phenol (*o*-cresol (#152)) and one furanic compound (2-pentylfuran (35)), were found.

**Figure 4:**
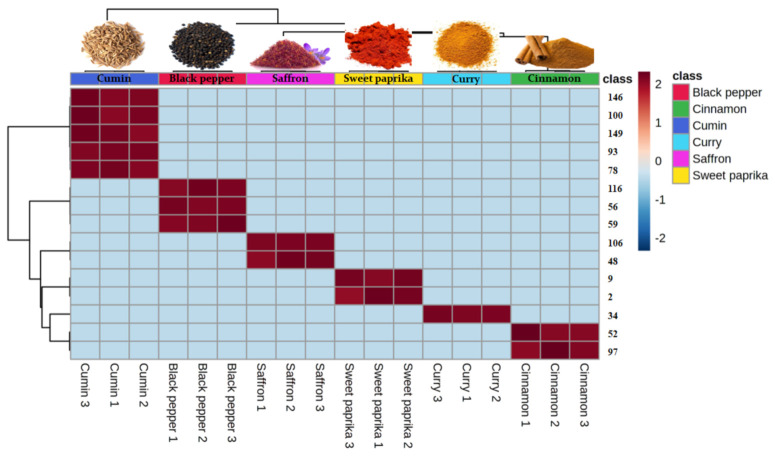
Hierarchical cluster analysis (HCA) and heat map from the dataset of volatile compounds were performed using the 15 most significant volatile organic metabolites (VOMs) identified in six spice samples (saffron, cinnamon, cumin, curry, black pepper and sweet paprika) obtained by ANOVA. The columns in the heatmap represent the cases (samples) and the rows indicate the variables (VOMs). The color gradient ranging from dark blue through white to dark red indicates the relationship of each variable to the sample, which represents the low, middle and high abundance of a VOM, respectively. The resulting dendrogram associated with the heatmap was generated by Ward’s algorithm and Euclidean distance analysis. Variable numbers: 146: nerolidol; 100: α-guaiene; 149: germacrene; 93: myrtenal; 78: pinocamphone; 116: b-selinene; 56: α-cububene; 59: β-pathoulene; 106: β-panasinsense; 48: 3-methyl styrene; 9: 3-methyl butanal; 2: 2,4-dymethyl heptane; 34: 2-hexenal; 52: β-ionone; 97: acetophenone.

The obtained *p*_values_ by one-way ANOVA with Fisher post-hoc test (*p* < 0.001) indicated that 94 of 162 VOMs identified were significantly different among the investigated spices. Moreover, HCA was also performed using the 15 most significant VOMs identified in the spice samples obtained by ANOVA. The resulting dendrogram associated with the heat map was performed by Euclidean distance through Ward’s clustering method (Figure 4), providing intuitive visualization of the data set, which along with the statistical analyses carried out previously, allows better identification of the inherent clustering patterns between each spice. Furthermore, these chemometric analyses are an excellent tool for authentication and quality control of the investigated spices.

## 3. Materials and Methods

### 3.1. Chemical and Reagents

Ultrapure water was obtained from a Milli-Q^®^ system (Millipore, Bedford, MA, USA). Internal standard (IS) 3-octanol and sodium chloride (NaCl, 99.5%) were obtained from Sigma-Aldrich (Madrid, Spain), whereas the GC carrier gas, helium of purity 5.0 was obtained from Air Liquide, Portugal. The SPME fiber coated with divinylbenzene/carboxen/polydimethylsiloxane (DVB/CAR/PDMS) (50/30 µm), SPME holder for manual sampling and glass vials were purchased from Supelco (Bellefonte, PA, USA). The alkane series, C8 to C20, with a concentration of 40 mg/L in *n*-hexane used to determine the kovats index (KI) was supplied from Fluka (Buchs, Switzerland).

### 3.2. Spice Samples

Five types of spices were selected for analysis i.e., saffron (*Crocus sativus* L.), cinnamon (*Cinnamomum verum*), cumin (*Cuminum cyminum* L.), black pepper (*Piper nigrum* L.) and sweet paprika (*Capsicum annuum* L.) and curry, a mixture of herbs and spices. A total of 18 samples, 3 packages per spice type, were collected in retail outlets in Funchal (Madeira Island, Portugal). There was no information available about how these spices were processed. The spices were purchased already powdered, packed in plastic bags, and stored in the dark at room temperature until analysis by HS-SPME/GC-qMS.

### 3.3. HS-SPME Procedure

A time-effective and solventless (HS-SPME) method was applied for VOMs extraction. With slight modifications, the HS-SPME procedure was performed based on the conditions described by Figueira et al. [22]. For headspace sampling, 1 g of spice powder, 0.3 g of NaCl (to promote the “salting-out” effect by decreasing the solubility of volatile metabolites in the water-based phase) and 6 mL of ultra-pure Milli-Q water were placed into a 20 mL amber headspace glass vial containing a magnetic stirring microbar. Before sealing the vial with a PTFE-faced silicone septum, 5 μL of 3-octanol (102 μg/mL) used as an internal standard was added. Then, the vial was placed in a thermostatic bath at 45 ± 1 °C under constant magnetic stirring (450 rpm). HS-SPME extractions were carried out by exposing the SPME fiber (DVB/CAR/PDMS) to the sample’s headspace for 50 min. Finally, the fiber was withdrawn into the holder needle, removed from the vial, and the VOMs extracted by SPME were thermally desorbed by the direct insertion of the fiber into a GC injector at 250 °C for 6 min, in splitless mode. Experiments were carried out in triplicate (*n* = 3) for all samples. The SPME fiber was thermally conditioned according to the manufacturer’s instructions before use, and a daily conditioning for 10 min was carried out before the first extraction to ensure the absence of carryover.

### 3.4. Fingerprinting of Spices by GC-MS Analysis

The fingerprint analysis of the investigated spices was performed using an Agilent Technologies 6890N (Palo Alto, CA, USA) gas chromatography system. This was equipped with a SUPELCOWAX^®^ 10 fused silica capillary column (60 m × 0.25 mm i.d. × 0.25 µm film thickness) supplied by Supelco (Bellefonte, PA, USA), with helium (Helium N60, Air Liquid, Portugal) as a carrier gas at a flow rate of 1 mL/min (column-head pressure: 13 psi). The injector temperature was fixed at 250 °C and a splitless injector equipped with an insert of 0.75 mm i.d. was attached. The oven temperature program was run as follows: initial temperature 40 °C for 1 min, 2.5 °C/min ramp until 220 °C and then held isothermally at 220 °C for 10 min, for a total GC run time of 83 min. MS detection was performed in full scan in an Agilent 5975 quadrupole inert mass selective detector (Santa Clara, CA, USA), the ion energy used for the electron impact (EI) was 70 eV and the source temperature was 230 °C. The electron multiplier was set to the autotune procedure. The mass acquisition range, made in full scan mode, was 30–300 m/z. VOMs were identified based on their mass spectra compared with those in the National Institute of Standards and Technology (NIST) MS 05 spectral database (Gaithersburg, MD, USA) with a matching probability > 85%, and determining the Kovat’s retention indices (RI) of each identified VOM according to the van den Dool and Kratz [47] equation: RI_x_ = 100n + 100(t_x_ − t_n_)(t_n + 1_ − t_n_), where t_n_ and t_n + 1_ are the retention times of the reference *n*-alkane hydrocarbons eluting immediately after and before the compound “x”, and t_x_ is the retention time of compound ‘x’. A C_8_–C_20_ saturated *n*-alkane solution was used to determine the RI, and the values were compared, when available, to values reported in the literature for similar columns [48,49,50,51] and databases available online (the Pherobase and Flavornet). Each sample was analyzed in triplicate.

### 3.5. Data Treatment and Multivariate Statistical Analysis

MSDA was performed using the MetaboAnalyst 5.0 web-based tool [52]. The raw GC-qMS data were pre-processed to remove the VOMs with missing values and then normalized (data transformation by cubic root and data scaling by autoscaling). The data matrix is subjected to a one-way analysis of variance (ANOVA) followed by Fisher’s test for post hoc multiple comparisons of means from the six spice varieties data at *p*-value < 0.001 to identify significant differences. The principal component analysis (PCA) and partial least squares-discriminant analysis (PLS-DA) were used to provide insights into the separations among the spices under study, and to detect the VOMs that may represent differences among the sample sets. Importantly, PLS-DA can identify VOMs sets that best discriminate among the different spices analyzed by reducing the size of the data matrix through eliminating redundant variables. The VOMs with variable importance in the projection (VIP) score ≥ 1.45 and differentially expressed in the univariate analysis were potential candidates for characterizing spice varieties. A hierarchical cluster analysis (HCA) was carried out using the 15 most significant VOMs identified in the spice samples obtained by ANOVA. This was generated through Ward’s algorithm and Euclidean distance analysis, aiming to identify clustering patterns for the characterization of the spices analyzed. Significance was established at *p* < 0.05.

## 4. Conclusions

The volatilomic composition of spices obtained by volatiles extraction through HS-SPME_DVB/CAR/PDMS_ followed by GC-qMS analysis revealed a useful strategy for the quality and authenticity purposes of spices. For each investigated spice, the most dominant chemical classes (terpenoids, sesquiterpenoids and carbonyl compounds) and major VOMs (dihydrojuneol, #161, in saffron; cinnamaldehyde, #150, in cinnamon; cuminaldehyde, #130, in cumin and curry; caryophyllene, #87, in black pepper; and *p*-cymene, #42, in sweet paprika), were determined and established. In addition, the VOMs common to all spices under study, and the VOMs identified only in one spice type were also determined. Even though all of the spices share the plant-related VOMs groups, the combination of both chromatography techniques and chemometrics analysis on the volatile analysis of the spices, resulted in a deep comprehensive characterization of the spices. This can be useful for the authentication analysis, helping with the detection of frauds and adulterations. In addition, some VOMs are part of biologically active compounds and have a high influence on the spice’s bioactive potential. This helps in the prevention of certain diseases including cancer, inflammatory-related diseases, diabetes, and cardiovascular diseases.

## Figures and Tables

**Table 1:** Relative area of volatile organic metabolites (VOMs) identified in spices using HS-SPME_DVB/CAR/PDMS_/GC-qMS.

Peak n°	RT ^a^ (min)	KI_cal_^b^	KI_lit_^c^	Volatile Metabolites	MF ^d^	Chem. Family	Relative Area (% RSD)					
							Saffron	Cinnamon	Cumin	Curry	Black Pepper	Sweet Paprika
1	8.134	775	768	4-Methylheptane	C_8_H_18_	HC	-	-	-	-	-	0.16 (16)
2	8.934	808	797	2,4-Dimethylheptane	C_9_H_20_	HC	-	-	-	-	-	0.14 (6)
3	9.273	823	821	Acetone	C_3_H_6_O	CC	-	-	-	-	-	0.26 (4)
4	10.122	857	852	4-Methyloctane	C_9_H_20_	HC	-	-	-	-	-	0.19 (12)
5	10.696	878	877	2-Methylfuran	C_5_H_6_O	FC	-	-	-	-	-	0.08 (16)
6	10.879	885	885	2,4-Dimethyl-1-heptene	C_9_H_18_	HC	-	-	-	-	-	0.62 (18)
7	11.083	892	892	Ethyl acetate	C_4_H_8_O_2_	E	29.10 (8)	-	-	-	-	-
8	12.119	923	931	2-Methyl-butanal	C_5_H_10_O	CC	-	-	-	-	-	0.27 (8)
9	12.267	927	936	3-Methyl-butanal	C_5_H_10_O	CC	-	-	-	-	-	0.34 (3)
10	12.731	939	941	Ethyl alcohol	C_2_H_6_O	A	2.94 (24)	5.81 (23)	11.64 (7)	-	0.77 (10)	0.38 (47)
11	13.450	957	957	2,2,4,6,6-Pentamethyl-heptane	C_12_H_26_	HC	-	0.90 (23)	-	-	-	0.63 (5)
12	13.650	962	962	2-Ethylfuran	C_6_H_8_O	FC	-	-	-	-	-	0.07 (23)
13	15.035	994	982	Methyl butyrate	C_5_H_10_O_2_	E	-	-	-	-	-	-
14	15.269	999	1005	4-Methyl decane	C_11_H_24_	HC	-	-	-	-	-	0.39 (9)
15	16.679	1028	1028	α-Pinene	C_10_H_16_	T	6.19 (9)	0.55 (18)	58.06 (14)	5.36 (17)	167.48 (18)	0.35 (8)
16	16.868	1032	1032	α-Thujene	C_10_H_16_	T	0.74 (6)	-	20.84 (12)	1.06 (15)	4.95 (17)	0.21 (25)
17	17.826	1050	1050	Toluene	C_7_H_8_	HC	12.05 (13)	-	-	2.24 (9)	1.29 (13)	-
18	18.513	1063	1064	α-Fenchene	C_10_H_16_	T	-	-	-	-	1.26 (8)	-
19	18.906	1070	1070	Camphene	C_10_H_16_	T	-	-	4.04 (14)	0.75 (16)	6.11 (10)	-
20	20.042	1089	1088	Hexanal	C_6_H_12_O	CC	-	-	-	0.38 (3)	-	0.78 (8)
21	20.840	1108	1109	2-Methyl-2-butenal	C_5_H_8_O	CC	-	-	-	1.92 (5)	-	-
22	20.794	1107	1105	β-Pinene	C_10_H_16_	T	3.11 (11)	0.92 (23)	1432.11 (10)	40.10 (14)	481.48 (7)	0.80 (19)
23	21.546	1121	1121	Sabinene	C_10_H_16_	T	0.69 (9)	-	94.76 (10)	2.02 (15)	20.54 (5)	0.60 (6)
24	22.069	1130	1128	4-Carene	C_10_H_16_	T	1.11 (15)	-	-	-	1.48 (17)	-
25	23.197	1150	1140	(+)-3-Carene	C_10_H_16_	T	10.50 (6)	-	5.71 (11)	0.59 (19)	1292.60 (1)	0.54 (32)
26	23.424	1154	1154	1-Methyl pyrrole	C_5_H_7_N	NC	-	-	-	-	-	0.76 (25)
27	24.008	1164	1160	β-Myrcene	C_10_H_16_	T	5.28 (6)	-	113.03 (10)	11.39 (8)	193.81 (8)	0.42 (9)
28	24.297	1168	1168	α-Phellandrene	C_10_H_16_	T	121.53 (10)	-	9.26 (10)	10.73 (14)	270.18 (1)	0.27 (5)
29	25.161	1182	1182	Cumene	C_9_H_12_	T	-	-	1.23 (12)	0.25 (20)	-	-
30	25.199	1183	1183	α-Terpinene	C_10_H_16_	T	4.26 (8)	-	4.77 (9)	1.47 (4)	10.14 (11)	0.50 (14)
31	26.397	1201	1202	Limonene	C_10_H_16_	T	17.77 (6)	0.69 (18)	37.73 (10)	9.19 (11)	1217.49 (2)	1.76 (14)
32	27.087	1214	1212	β-Phellandrene	C_10_H_16_	T	6.77 (22)	-	38.71 (10)	2.75 (5)	45.95 (2)	0.49 (10)
33	27.229	1217	1217	Eucalyptol	C_10_H_18_O	T	49.35 (17)	1.12 (10)	28.08 (23)	2.65 (17)	-	-
34	28.224	1234	1234	2-Hexenal	C_6_H_10_O	CC	-	-	-	0.40 (1)	-	-
35	28.528	1240	1241	2-Pentylfuran	C_9_H_14_O	FC	-	-	-	-	-	0.45 (12)
36	29.355	1254	1254	γ-Terpinene	C_10_H_16_	T	4.79 (6)	1.01 (22)	2824.09 (7)	-	-	2.19 (5)
37	29.564	1257	1257	β-Ocimene isomer	C_10_H_16_	T	0.54 (23)	-	-	0.63 (6)	1.92 (5)	0.20 (18)
38	29.140	1250	1250	β-Phellandrene	C_10_H_16_	T	-	-	-	-	8.87 (3)	-
39	29.713	1260	1261	γ-Terpinene	C_10_H_16_	T	-	-	-	115.03 (12)	20.46 (5)	-
40	29.613	1258	1259	α-Ocimene	C_10_H_16_	T	-	-	-	-	6.03 (6)	-
41	30.476	1272	1275	Styrene	C_8_H_8_	HC	0.26 (22)	13.38 (13)	-	-	0.75 (9)	0.08 (23)
42	31.088	1282	1284	*p*-cymene	C_10_H_14_	T	78.13 (12)	1.72 (21)	1136.25 (6)	109.60 (9)	234.45 (3)	2.65 (16)
43	31.738	1293	1293	Terpinolene	C_10_H_16_	T	37.49 (12)	-	4.19 (7)	4.54 (23)	117.72 (17)	0.25 (20)
44	32.062	1298	1298	Octanal	C_8_H_16_O	CC	1.96 (14)	-	-	-	-	-
45	34.297	1337	1336	2,2,6-Trimethylcyclohexanone	C_9_H_16_O	T	-	-	-	-	-	0.23 (23)
46	34.571	1342	1340	2-Heptenal isomer	C_7_H_12_O	CC	-	-	2.72 (10)	-	-	-
47	35.088	1351	1342	6-Methyl-5-hepten-2-one	C_8_H_14_O	CC	1.06 (10)	3.95 (5)	-	0.73 (7)	-	0.50 (11)
48	36.638	1377	1385	3-Methylstyrene	C_9_H_10_	HC	0.50 (5)	-	-	-	-	-
49	36.718	1378	1372	Allyl isothiocyanate	C_4_H_5_NS	SC	-	-	-	17.63 (8)	-	-
50	37.959	1398	1400	Tetradecane	C_14_H_30_	HC	-	0.58 (11)	-	0.90 (1)	-	-
51	39.109	1419	1414	Fenchone	C_10_H_16_O	T	-	-	-	-	0.62 (12)	-
52	40.632	1446	1431	3-Ethyl-o-xylene	C_10_H_14_	HC	-	0.91 (6)	-	-	-	-
53	41.116	1454	1463	β-Ionone	C_13_H_20_O	T	-	7.51 (9)	-	-	-	-
54	41.138	1455	1456	Dehydro-p-cymene	C_10_H_14_	T	13.29 (11)	-	4.66 (8)	3.54 (8)	19.98 (16)	-
55	41.246	1457	1456	1,2,3,4-Tetramethyl-benzene	C_10_H_14_	HC	-	1.04 (7)	-	-	-	-
56	42.107	1471	1467	α-Cubebene	C_15_H_24_	ST	-	-	-	-	31.46 (2)	-
57	42.793	1483	1482	δ-Elemene	C_15_H_24_	ST	3.14 (13)	4.11 (16)	-	-	598.43 (16)	-
58	43.025	1487	1467	Tetramethyl-pyrazine	C_8_H_12_N_2_	NC	3.96 (16)	-	-	1.17 (3)	-	0.19 (10)
59	43.496	1495	1488	a-Patchoulene	C_15_H_24_	ST	-	-	-	-	3.51 (4)	-
60	43.815	1500	1491	Ylangene	C_15_H_24_	ST	-	9.28 (18)	-	-	1.22 (9)	-
61	43.901	1502	1492	Cyclosativene	C_15_H_24_	ST	-	51.44 (15)	-	-	5.74 (13)	-
62	44.240	1508	1509	Copaene	C_15_H_24_	ST	3.72 (14)	1064.64 (13)	60.09 (12)	5.63 (10)	386.46 (12)	0.35 (3)
63	45.528	1534	1531	β-Bourbonene	C_15_H_24_	ST	0.84 (24)	-	-	-	-	-
64	45.807	1539	1544	Calarene isomer	C_15_H_24_	ST	1.72 (22)	-	-	-	-	-
65	46.168	1546	1540	Camphor	C_10_H_16_O	T	-	-	3.88 (8)	23.57 (9)	5.76 (8)	0.14 (16)
66	46.308	1549	1562	α-Cedrene	C_15_H_24_	ST	1.03 (23)	-	-	-	-	-
67	46.424	1551	1558	Benzaldehyde	C_7_H_6_O	CC	3.76 (21)	85.40 (5)	-	-	-	0.88 (2)
68	46.491	1552	1552	Linalool	C_10_H_18_O	T	-	-	-	445.08 (10)	91.33 (9)	-
69	46.776	1557	1558	β cubebene	C_15_H_24_	ST	-	-	-	-	46.72 (19)	-
70	46.820	1558	1554	Calarene isomer	C_15_H_24_	ST	-	32.50 (14)	-	-	-	-
71	46.930	1560	1563	β-Terpineol	C_10_H_18_O	T	-	-	36.07 (9)	2.22 (22)	-	0.44 (25)
72	47.032	1562	1559	Isoledene	C_15_H_24_	ST	-	5.18 (13)	-	-	-	-
73	47.215	1566	1568	Linalyl acetate	C_12_H_20_O_2_	E	-	-	-	-	-	0.54 (21)
74	47.353	1568	1552	Aristolene	C_15_H_24_	ST	11.37 (7)	-	-	-	-	-
75	48.242	1585	1585	α-Bergamotene	C_15_H_24_	ST	-	9.54 (24)	-	-	-	-
76	48.561	1590	1597	α-Santalene	C_15_H_24_	ST	-	8.49 (16)	-	-	-	-
77	48.577	1591	1578	*p*-Menth-2-en-1-ol isomer	C_10_H_18_O	T	3.09 (13)	-	-	-	-	-
78	48.736	1593	1582	Pinocamphone	C_10_H_16_O	T	-	-	516.77 (3)	-	-	-
79	48.813	1595	1601	(-)-Clovene	C_15_H_24_	ST	2.26 (11)	17.26 (12)	-	-	-	-
80	48.972	1598	1602	a-Elemene	C_15_H_24_	ST	-	11.23 (21)	-	-	17.39 (13)	-
81	49.107	1600	1590	Bergamotene isomer	C_15_H_24_	ST	5.11 (3)	39.59 (17)	-	2.46 (6)	1.89 (16)	-
82	49.120	1600	1584	Isobornyl acetate	C_12_H_20_O_2_	E	-	-	47.68 (5)	-	-	-
83	49.554	1605	1605	β-Elmene	C_15_H_24_	ST	-	45.70 (14)	-	-	127.48 (16)	-
84	49.624	1606	1606	2-Undecanone	C_11_H_22_O	CC	2.72 (19)	-	-	-	-	-
85	50.032	1610	1619	(+)-Longifolene	C_15_H_24_	ST	-	6.43 (17)	-	-	-	-
86	50.060	1610	1606	(-)-Terpinen-4-ol	C_10_H_18_O	T	-	-	13.46 (3)	2.66 (9)	10.13 (14)	0.61 (4)
87	50.303	1613	1612	Caryophyllene isomer	C_15_H_24_	ST	123.13 (7)	37.67 (17)	59.95 (3)	9.71 (9)	3157.13 (13)	0.64 (15)
88	50.794	1618	1625	(+)-Aromadendrene	C_15_H_24_	ST	-	4.98 (18)	-	-	-	-
89	50.953	1620	1626	α-Cedrene	C_15_H_24_	ST	-	3.35 (19)	-	-	-	-
90	51.173	1622	1621	cis-p-2-menthen-1-ol	C_10_H_18_O	T	-	-	5.82 (8)	-	-	-
91	51.274	1623	1635	γ-Gurjunene	C_15_H_24_	ST	-	3.24 (23)	-	-	-	-
92	51.540	1626	1624	β-Cyclocitral	C_10_H_16_O	T	-	-	-	-	-	0.31 (6)
93	52.138	1632	1634	Myrtenal	C_10_H_14_O	T	-	-	6.09 (1)	-	-	-
94	52.610	1637	1653	(-)-β-Santalene	C_15_H_24_	ST	-	11.83 (22)	-	-	18.37 (4)	-
95	52.683	1637	1640	Pinocarveol	C_10_H_16_O	T	-	-	7.45 (6)	-	-	-
96	52.870	1639	1633	Farnesene isomer	C_15_H_24_	ST	34.21 (9)	-	78.79 (5)	4.43 (14)	17.53 (7)	-
97	53.172	1642	1645	Acetophenone	C_8_H_8_O	CC	-	73.03 (7)	-	-	-	-
98	53.189	1643	1640	β-Patchoulene	C_15_H_24_	ST	-	-	8.13 (9)	-	-	-
99	53.320	1644	1652	α-Humulene	C_15_H_24_	ST	-	-	-	0.98 (6)	6.74 (13)	-
100	53.723	1648	1652	α-Guaiene	C_15_H_24_	ST	-	-	12.04 (5)	-	-	-
101	53.774	1648	1652	Estragole	C_10_H_12_O	T	-	-	-	-	17.26 (7)	-
102	53.857	1649	1662	Pulegone	C_10_H_16_O	T	-	-	-	1.02 (1)	-	-
103	53.920	1650	1654	β-Humulene	C_15_H_24_	ST	-	7.15 (14)	-	-	-	-
104	54.063	1651	1672	Epizonarene	C_15_H_24_	ST	74.63 (5)	55.35 (18)	6.78 (3)	3.21 (2)	264.01 (15)	-
105	54.455	1704	1697	Farnesene isomer	C_15_H_24_	ST	7.06 (13)	-	-	-	-	-
106	54.586	1707	1689	β-Panasinsene	C_15_H_24_	ST	24.62 (1)	-	-	-	-	-
107	54.755	1710	1710	α-Terpineol	C_10_H_18_O	T	6.48 (18)	-	21.55 (7)	5.15 (5)	49.27 (14)	0.26 (15)
108	54.812	1712	1725	γ-Muurolene	C_15_H_24_	ST	-	227.05 (20)	-	-	-	-
109	55.011	1716	1720	β-Cadinene	C_15_H_24_	ST	-	-	-	-	11.22 (9)	-
110	55.120	1718	1707	Zonarene	C_15_H_24_	ST	-	-	78.06 (5)	5.23 (10)	-	-
111	55.256	1721	1721	γ-Himachalene	C_15_H_24_	ST	-	29.13 (23)	-	-	-	-
112	55.287	1721	1726	Valencene	C_15_H_24_	ST	-	-	-	-	15.33 (14)	-
113	55.336	1722	1731	(-)-Zingiberene	C_15_H_24_	ST	-	-	37.51 (6)	2.00 (13)	-	-
114	55.905	1734	1736	Bicyclogermacrene	C_15_H_24_	ST	-	81.26 (23)	-	-	-	-
115	56.093	1738	1749	(-)-δ-Cadinene	C_15_H_24_	ST	450.35 (7)	-	-	16.82 (10)	-	-
116	56.109	1738	1731	α-Selinene	C_15_H_24_	ST	-	-	-	-	70.59 (4)	-
117	56.386	1744	1741	β-Bisabolene	C_15_H_24_	ST	134.58 (6)	109.30 (24)	14.80 (12)	5.11 (11)	26.80 (15)	0.28 (1)
118	56.543	1747	1750	α-Muurolene	C_15_H_24_	ST	-	473.10 (23)	-	-	8.15 (5)	-
119	56.737	1751	1742	β-Selinene	C_15_H_24_	ST	-	-	-	-	102.21 (16)	-
120	56.828	1753	1733	Citral isomer	C_10_H_16_O	T	-	-	-	-	-	0.40 (17)
121	56.858	1753	1779	γ-Selinene	C_15_H_24_	ST	-	37.63 (21)	-	-	69.06 (17)	-
122	56.922	1754	1759	Phellandral	C_10_H_16_O	T	-	-	97.64 (4)	3.14 (14)	-	-
123	57.037	1757	1760	(+)-Epi-bicyclosesquiphellandrene	C_15_H_24_	ST	25.14 (15)	-	-	-	-	-
124	57.317	1762	1778	Germacrene B	C_15_H_24_	ST	-	-	-	-	18.87 (13)	-
125	57.45	1765	1765	Citronellol	C_10_H_20_O	T	-	-	-	26.91 (10)	-	-
126	58.177	1779	1749	δ-Cadinene	C_15_H_24_	ST	-	717.65 (24)	-	-	197.13 (12)	-
127	58.551	1787	1786	Bisabolene isomer	C_15_H_24_	ST	-	68.37 (24)	-	-	12.53 (15)	-
128	58.662	1789	1782	β-Sesquiphellandrene	C_15_H_24_	ST	511.76 (10)	-	3.91 (9)	18.70 (9)	-	-
129	58.812	1792	1777	α-Curcumene	C_15_H_22_	ST	817.86 (8)	80.41 (22)	4.48 (18)	27.17 (8)	7.76 (8)	-
130	59.432	1804	1802	Cuminaldehyde	C_10_H_12_O	T	27.33 (14)	5.69 (24)	9694.84 (3)	777.58 (10)	24.54 (15)	1.40 (20)
131	59.548	1807	1813	Methyl salicylate	C_8_H_8_O_3_	E	-	21.04 (23)	-	-	12.70 (13)	-
132	59.972	1817	1840	α-Panasinsene	C_15_H_24_	ST	-	19.58 (20)	-	-	-	-
133	60.283	1824	1821	Neryl isobutyrate	C_14_H_20_O_2_	E	-	-	-	-	22.10 (14)	-
134	60.287	1824	1847	*Cis*-carveol	C_10_H_16_O	T	8.86 (15)	-	-	-	-	-
135	61.508	1851	1851	Geraniol	C_10_H_18_O	T	9.87 (19)	-	-	-	-	1.06 (21)
136	61.561	1853	1845	Anethole	C_10_H_12_O	T	-	5.08 (25)	98.43 (9)	10.09 (3)	5.58 (15)	-
137	61.705	1856	1868	Thymol acetate	C_12_H_16_O_2_	E	-	-	-	-	7.23 (15)	-
138	61.978	1862	1859	Calamenene	C_15_H_22_	ST	-	420.90 (24)	-	-	26.87 (13)	-
139	62.036	1863	1861	2,4-Dimethylacetophenone	C_10_H_12_O	CC	27.51 (15)	-	-	2.84 (21)	-	-
140	63.653	1898	1902	Geranyl butyrate	C_14_H_24_O_2_	E	25.14 (9)	-	-	2.55 (16)	-	-
141	63.944	1905	1903	Safrole	C_10_H_10_O_2_	T	-	-	-	-	2.31 (8)	-
142	65.943	1951	1926	α-Calacorene	C_15_H_20_	ST	-	94.52 (24)	-	-	3.87 (3)	-
143	66.413	1962	1962	2-(4-Methylphenyl)propan-1-ol	C_10_H_14_O	A	-	-	-	-	10.56 (12)	-
144	66.688	1968	1975	Jasmone	C_11_H_16_O	T	-	-	-	-	-	0.52 (15)
145	67.640	1990	1996	4-Methylguaiacol	C_8_H_10_O_2_	VP	-	-	-	2.28 (17)	-	-
146	68.058	1999	2009	Nerolidol	C_15_H_26_O	ST	-	-	12.66 (3)	-	-	-
147	69.278	2009	2008	Caryophyllene oxide	C_15_H_24_O	ST	29.20 (11)	-	-	-	84.25 (12)	-
148	69.449	2010	2012	Methyl eugenol	C_11_H_14_O_2_	VP	-	146.68 (23)	121.21 (46)	-	-	-
149	71.220	2023	2018	Germacrene D-4-ol	C_15_H_26_O	ST	-	-	58.67 (4)	-	-	-
150	71.667	2026	2017	Cinnamaldehyde	C_9_H_8_O	CC	-	6996.90 (24)	84.27 (41)	-	7.77 (9)	1.09 (11)
151	72.319	2030	2036	Cadinol	C_15_H_26_O	ST	12.26 (16)	-	-	-	-	-
152	72.429	2031	2033	o-Cresol	C_7_H_8_O	VP	-	-	-	-	6.39 (11)	-
153	73.253	2137	2143	γ-Eudesmol	C_15_H_26_O	ST	-	-	389.01 (4)	26.61 (8)	-	-
154	74.492	2145	2166	Spathulenol	C_15_H_24_O	ST	-	-	-	-	4.71 (10)	-
155	75.544	2152	2150	Eugenol	C_10_H_12_O_2_	VP	-	-	23.05 (13)	-	-	1.93 (20)
156	77.911	2168	2157	Thymol	C_10_H_14_O	VP	-	-	26.30 (18)	-	7.52 (13)	1.64 (6)
157	77.914	2168	2173	Carvacrol	C_10_H_14_O	VP	16.23 (16)	-	-	-	-	-
158	78.182	2170	2175	α-Bisabolol	C_15_H_26_O	ST	-	14.02 (22)	-	-	-	-
159	79.296	2177	2177	τ-Muurolol	C_15_H_26_O	ST	-	8.19 (21)	-	-	-	-
160	79.587	2179	2188	Cadalene	C_15_H_18_	ST	-	31.97 (24)	-	-	-	-
161	80.607	2185	2187	Dihydrojuneol	C_15_H_28_O	ST	1447.25 (8)	-	-	92.27 (15)	-	-
162	81.898	2193	2209	Cadinol	C_15_H_26_O	ST	-	19.41 (21)	-	-	-	-

HC: Hydrocarbon; CC: Carbonyl compound; FC: Furanic compound; E: Ester; A: Alcohol; T: Terpenoid; NC: Nitrogen compound; SC: Sulfur compound; ST: Sesquiterpenoid; VP: Volatile phenol; -: Not detected. ^a^ RT: Retention time; ^b^ Kovat index relative *n*-alkanes (C_8_ to C_20_) on a BP-20 capillary column; ^c^ Kovat index relative reported in the literature for equivalent capillary column [26]; ^d^ MF: Molecular formula.

**Table 2:** Potential bioactive effects of some important VOMs identified in the studied spices.

Peak n°	Volatile Organic Metabolites	Potential Bioactive Effects ^1^								Spices	References
		Antibacterial	Antidiabetic	Anti-inflammatory	Antifungal	Antioxidant	Antiproliferative	Antitumor	Cytotoxic		
22	β-Pinene				x	x	x	x		Saffron, cinnamon, cumin, curry, black pepper and sweet paprika	[4,11,33,39,40]
25	(+)-3-Carene	x				x		x		Saffron, cumin, curry, black pepper and sweet paprika	
27	β-Myrcene		x	x	x	x	x	x	x	Saffron, cumin, curry, black pepper and sweet paprika	
31	Limonene	x	x	x	x	x	x	x	x	Saffron, cinnamon, cumin, curry, black pepper and sweet paprika	
36	γ-Terpinene	x			x			x	x	Saffron, cinnamon, cumin, curry, black pepper and sweet paprika	
42	*p*-Cymene	x		x	x	x			x	Saffron, cinnamon, cumin, curry, black pepper and sweet paprika	
68	Linalool	x	x	x		x	x			Curry and black pepper	
78	Pinocamphone	x			x	x		x	x	Cumin	
87	Caryophyllene isomer	x	x			x	x		x	Saffron, cinnamon, cumin, curry, black pepper and sweet paprika	
118	α-Muurolene							x	x	Cinnamon and black pepper	
128	β-Sesquiphellandrene	x	x	x		x			x	Saffron, cumin and curry	
129	α-Curcumene			x				x		Saffron, cinnamon, cumin, curry and black pepper	
130	Cuminaldehyde	x	x	x	x			x	x	Saffron, cinnamon, cumin, curry, black pepper and sweet paprika	
150	Cynamaldehyde	x	x	x		x		x	x	Cinnamon, cumin, black pepper and sweet paprika	
155	Eugenol	x				x		x		Cumin and sweet paprika	
156	Thymol	x		x	x	x				Cumin, black pepper and sweet paprika	

^1^ Potential bioactive effect indicates the type of bioactive effects reported for each of the volatile organic metabolites (VOMs) referred in Table 1.

## Data Availability

All the data in this research are presented in manuscript and supplementary material.

## Supplementary Materials

The following supporting information can be downloaded at: https://www.mdpi.com/article/10.3390/molecules27196403/s1, Figure S1: Chromatogram of the volatile fraction of the spice samples obtained by HS-SPME-GC-qMS. The numbers above the peaks indicate the volatile organic metabolites (VOMs) identified in Table 1. Peak number identification: 15—α-pinene, 22—β-pinene, 23—sabinene, 25—3-carene, 27—β-myrcene, 28—α-phellandrene, 31—limonene, 33—eucalyptol, 36—terpinene isomer, 39—terpinene isomer, 42—*p*-cymene, 43—terpinolene, 57—δ-elemene, 62—copaene, 67—benzaldehyde, 68—linalool, 78—pinocamphone, 83—β-elemene, 86—terpinen-4-ol, 87—caryophyllene isomer, 104—epizonarene, 108—γ-muurolene, 114—bicyclogermacrene, 115—(-)-δ-cadinene, 116—selinene isomer, 117—β-bisabolene, 118—α-muurolene, 119—selinene isomer, 126—δ-cadinene, 128—β-sesquiphellandrene, 129—α-curcumene, 130—cuminalgehyde, 135—geraniol, 138—calamenene, 142—α-calacorene, 147—caryophyllene oxide, 150—cinnamaldehyde, 153—γ-eudesmol, 155—eugenol, 156—thymol, 161—dihydrojuneol.; Table S1: Volatile organic metabolites (VOMs) identified only in a determined spice using HS-SPME_DVB/CAR/PDMS_/GC-qMS.

## Sample Availability

Samples of the compounds are available from the authors.
