# Supplementary material for: Spices Volatilomic Fingerprinting—A Comprehensive Approach to Explore Its Authentication and Bioactive Properties
Source: Molecules. 2022 Sep 28;27(19):6403. doi: 10.3390/molecules27196403 (PMC9570555; doi:10.3390/molecules27196403)
Supplement: Supplementary file 1 [file molecules-27-06403-s001.zip › molecules-1939502-supplementary.pdf]

Spices volatilomic fingerprinting – a comprehensive approach to explore its authentication and bioactive properties

Sergio Izcara[1,2], Rosa Perestrelo[2], Sonia Morante-Zarcero[1], Isabel Sierra[1] and José S. Câmara[2,3,*]

[1] *Departamento de Tecnología Química y Ambiental, E.S.C.E.T, Universidad Rey Juan Carlos, C/ Tulipán s/n, 28933 Móstoles, Madrid, Spain*
[2] *CQM – Centro de Química da Madeira, Universidade da Madeira, Campus da Penteada, 9020-105 Funchal, Portugal*
[3] *Departamento de Química, Faculdade de Ciências Exatas e Engenharia, Universidade da Madeira, Campus da Penteada, 9020-105 Funchal, Portugal*

SUPPLEMENTARY MATERIAL

Table S1. Volatile organic metabolites (VOMs) identified only in a determined spice using HS-SPME$_{DVB/CAR/PDMS}$/GC-qMS.

**Saffron**	**Cinnamon**	**Cumin**	**Curry**	**Black pepper**	**Sweet paprika**
Ethyl acetate	3-Ethyl-o-xylene	2-Heptenal	2-Methyl-2-butenal	α-Fenchene	4-Methylheptane
Octanal	β-Ionone	Pinocamphone	2-Hexenal	β-Phellandrene	2,4-Dimethylheptane
3-Methyl styrene	TMB [a]	Isobornyl acetate	Allyl isothiocyanate	α-Ocymene	Acetone
β-Bourbonene	Isoledene	*p*-2-Menthen-1-ol	Pulegone	Fenchone	4-Methyloctane
calarene,	α-Bergamotene	Myrtenal	Citronellol	α-Cububene	2-Methylfuran
α-Cedrene	α-Santalene	Pinocarveol	4-Methyl-guaiacol	α-Patchoulene	DMH [b]
Aristolene	Longifolene	β-Patchoulene		β-cubebene	2-Methyl butanal
2-Undecanone	Aromadendrene	α-Guaiene		Estragole	3-Methyl butanal
Farnesene,	α-Cedrene	Nerolidol		β-Cadinene	1-Methyl pyrrole
β-Panasinsene	γ-Gurjunene	D-Germacrene		Valencene	2-Pentyl furan
cis-Carveol	β-Humulene			α-Selinene	TMCH [c]
Geraniol	γ-Muurolene			β-Selinene	Linalyl acetate
Cadinol	γ-Himachelene			Germacrene B	β-Cyclocitral
Carvacrol	Bicyclogermacrene			Neryl isobutyrate	Citral
	α-Panasinsense			Thymol acetate	Jasmone
	α-Bisabolol			Safrol	
	t-Muurolol			*o*-Cresol	
	Cadalene			Spathulenol	

[a] 1,2,3,4-Tetramethyl benzene; [b] 2,4-Dimethyl-1-heptene; [c] 2,6,6-teimethyl cyclohexanone

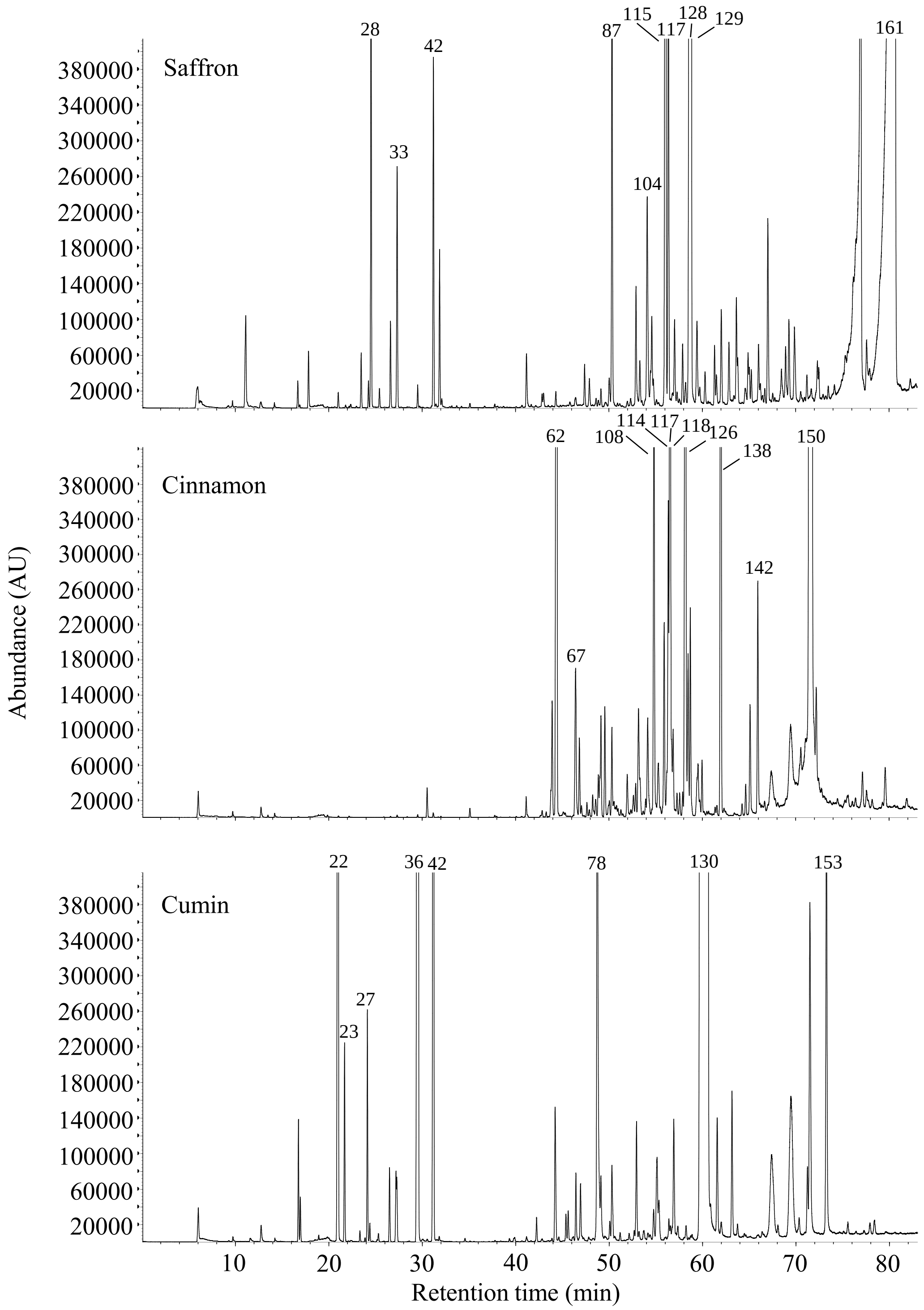

Saffron
28
33
42
87
104
115
117
128
129
161
Cinnamon
62
67
108
114
117
118
126
138
142
150
Cumin
22
23
27
36
42
78
130
153
380000
340000
300000
260000
220000
180000
140000
100000
60000
20000
Abundance (AU)
10
20
30
40
50
60
70
80
Retention time (min)

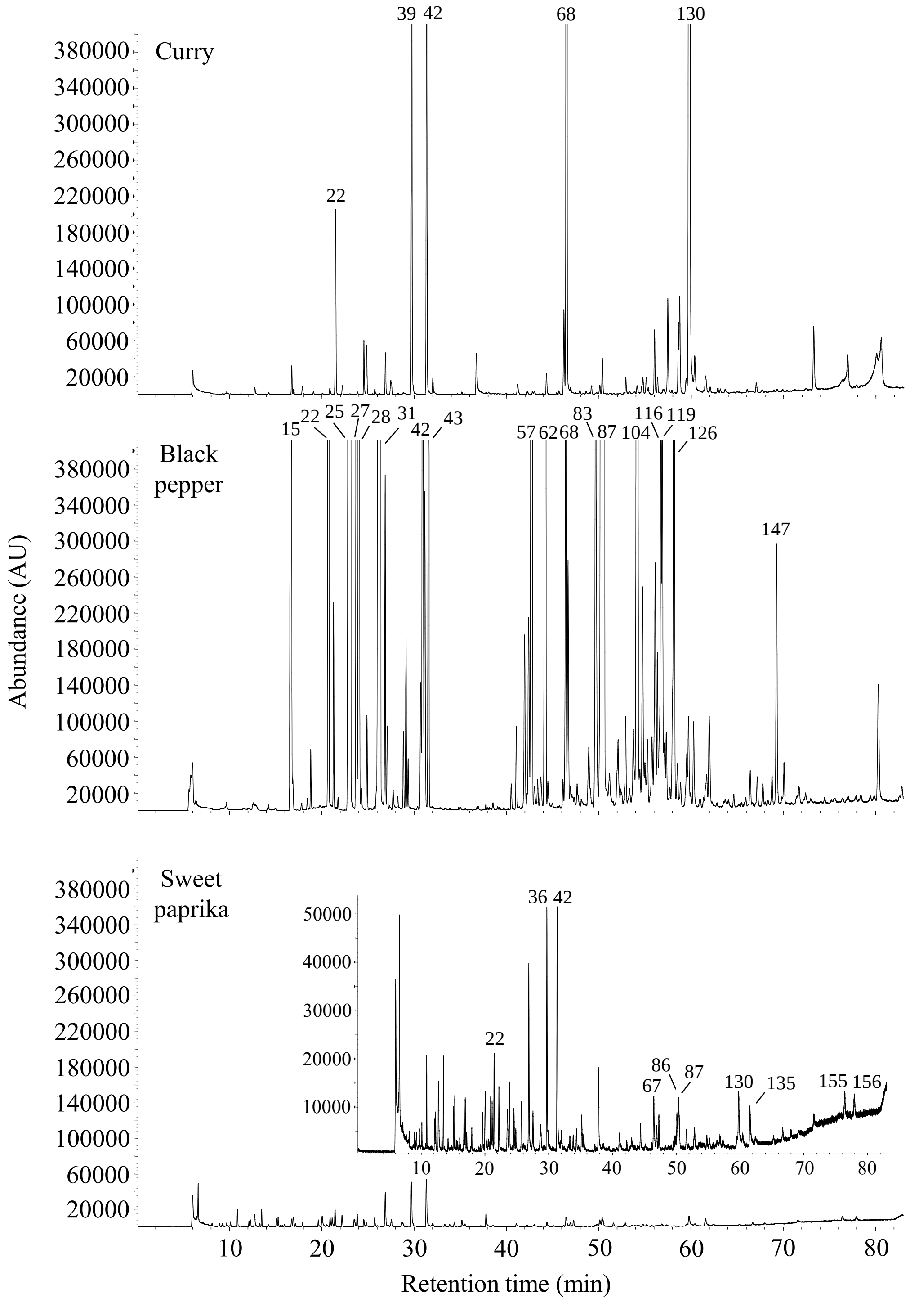

Figure S1. Chromatogram of the volatile fraction of the spice samples obtained by HS-SPME-GC-qMS. Numbers above the peaks indicate the volatile organic metabolites (VOMs) identified in Table 1. Peak number identification: 15 – α-pinene, 22 – β-pinene, 23 – sabinene, 25 – 3-carene, 27 – β-myrcene, 28 – α-phellandrene, 31 – limonene, 33 – eucalyptol, 36 – terpinene isomer, 39 – terpinene isomer, 42 – p-cymene, 43 – terpinolene, 57 – δ-elemene, 62 – copaene, 67 – benzaldehyde, 68 – linalool, 78 – pinocamphone, 83 – β-elemene, 86 – terpinen-4-ol, 87 – caryophyllene isomer, 104 – epizonarene, 108 – γ-muurolene, 114 – bicyclogermacrene, 115 – (-)-δ-cadinene, 116 – selinene isomer, 117 – β-bisabolene, 118 – α-muurolene, 119 – selinene isomer, 126 – δ-cadinene, 128 – β-sesquiphellandrene, 129 – α-curcumene, 130 – cuminalgehyde, 135 – geraniol, 138 – calamenene, 142 – α-calacorene, 147 – caryophyllene oxide, 150 – cinnamaldehyde, 153 – γ-eudesmol, 155 – eugenol, 156 – thymol, 161 – dihydrojuneol.
